# Fungal Diversity in Tomato Rhizosphere Soil under Conventional and Desert Farming Systems

**DOI:** 10.3389/fmicb.2017.01462

**Published:** 2017-08-02

**Authors:** Elham A. Kazerooni, Sajeewa S. N. Maharachchikumbura, Velazhahan Rethinasamy, Hamed Al-Mahrouqi, Abdullah M. Al-Sadi

**Affiliations:** Department of Crop Sciences, College of Agricultural and Marine Sciences, Sultan Qaboos University Muscat, Oman

**Keywords:** farming system, fungal community, pathogen, saprophytes, desert fungi

## Abstract

This study examined fungal diversity and composition in conventional (CM) and desert farming (DE) systems in Oman. Fungal diversity in the rhizosphere of tomato was assessed using 454-pyrosequencing and culture-based techniques. Both techniques produced variable results in terms of fungal diversity, with 25% of the fungal classes shared between the two techniques. In addition, pyrosequencing recovered more taxa compared to direct plating. These findings could be attributed to the ability of pyrosequencing to recover taxa that cannot grow or are slow growing on culture media. Both techniques showed that fungal diversity in the conventional farm was comparable to that in the desert farm. However, the composition of fungal classes and taxa in the two farming systems were different. Pyrosequencing revealed that *Microsporidetes* and *Dothideomycetes* are the two most common fungal classes in CM and DE, respectively. However, the culture-based technique revealed that Eurotiomycetes was the most abundant class in both farming systems and some classes, such as *Microsporidetes*, were not detected by the culture-based technique. Although some plant pathogens (e.g., *Pythium* or *Fusarium*) were detected in the rhizosphere of tomato, the majority of fungal species in the rhizosphere of tomato were saprophytes. Our study shows that the cultivation system may have an impact on fungal diversity. The factors which affected fungal diversity in both farms are discussed.

## Introduction

Soil is a precious and complex natural resource that represents a huge reservoir of biodiversity with several billion prokaryotic and eukaryotic microorganisms. These microbes significantly share biomass and ecosystem functions in both natural and managed agricultural soils (Sidorenko et al., 1978). Microbial diversity is directly or indirectly affected by cultivation techniques, management practices, crop rotation, soil tillage, animal grazing, plant species and climatic changes (Acosta-Martínez et al., 2014; Chen et al., 2017; Gangireddygari et al., 2017; Liu et al., 2017; Yao et al., 2017). Variations in soil temperature, precipitation and soil pH also influence soil fungal diversity. Fungi are the dominant eukaryotes among soil microbial communities where they play crucial and key roles in terrestrial ecosystems (Abed et al., 2013; Peay et al., 2013; Acosta-Martínez et al., 2014).

Oman is situated at an arid region in the eastern part of the Arabian Peninsula and in summer, the temperature can rise up to 50°C. Most farms in Oman use traditional methods to promote biodiversity by cultivating several crops in the same field. The majority of farms in the northern part of the country grow date palms, while rest occupies citrus, vegetable crops such as tomatoes and cucumbers and other crops (Kazeeroni and Al-Sadi, 2016). Tomato is the major vegetable crop produced in Oman with the total production of over 70,000 tons in 2014 (FAO, 2015). Most tomato production in Oman is in commercial farms in the main agricultural area, the Batinah region. However, some of the farms that are located in desert areas also produce crops including tomatoes.

Microbial abundance, diversity and activity largely have implications on sustainable productivity of agricultural land and production systems. Information on the microbial communities associated with rhizospheres and their complex interrelationship is essential in the selection of sustainable crop rotations and management practices (Lenc et al., 2015; Chen et al., 2017). Direct culture of microorganisms and molecular methods are widely used to analyze soil microbes (Al-Sadi et al., 2015; Thomson et al., 2015; Kazeeroni and Al-Sadi, 2016). With the advent of next generation sequencing technologies, 454 pyrosequencing is used nowadays for assessing fungal diversity because of its high sensitivity (Esmaeili Taheri et al., 2015; Kazeeroni and Al-Sadi, 2016).

Although several studies addressed tomatoes, the information about the occurrence and the organization of fungal organisms in the tomato rhizosphere is currently limited. Furthermore, studies on fungal diversity in desert farming systems remain rare. Considering the different ways that can change the farming systems underlying soils and soil microbes, it is essential to understand the fungal diversity and their functions in soils under different managements. In this study, we examined soil fungal composition and diversity using pyrosequencing and culture-based techniques in two different tomato-farming systems: commercial vs. desert. Our main objective was to study how the changes of soil fungal communities vary with the different farming techniques. Knowledge in these areas will help predict how fungal communities vary under varying cultivation systems.

## Materials and Methods

### Collection of Samples

Soil samples were collected from conventional and desert farms in Barka and Thumrait, Oman during June 2014 and the information on the details of the locations and weather conditions of the soils samples are mentioned in **Table 1**. Each soil was collected along random directions from three different lots of each tomato plant, approximately about 1 kg from each sample, taken from 10 to 12 cm depth near the active growing roots. The soil samples were kept in sterile plastic bags and brought to the laboratory. All samples were thoroughly homogenized before stored at 10°C.

**Table 1:** Physicochemical properties of soil samples.

Sample name	Soil texture	pH	EC (mS)	%TIC	%TOC	%N	P (mg kg^-1^)	K (mg kg^-1^)
CM	Sandy	8.0 a	1.28 b	5.27 a	3.464 a	0.056 a	5.076 a	61.876 a
DE	Loamy sand	7.8 a	7.72 a	4.13 a	2.768 a	0.020 b	3.272 b	45.639 b

*EC, electrical conductivity; TIC, total inorganic carbon; TOC, total organic carbon; N, nitrogen; P, phosphorus; and K; potassium. Values with the same letter in the same column are not significantly different from each other at *P* < 0.05 (Tukey’s Studentized Range test, SAS).*

### Soil Analysis

Soil samples were air-dried and sieved. Soil texture, pH and electrical conductivity (EC) were determined using standard methods (Gee and Bauder, 1986; Zhang et al., 2005). Determination of potassium (K) and phosphorus (P) were done using a flame photometric method (Sheerwood 450 flame photometer) and Inductively Coupled Plasma (Perkin Elmer, United States), respectively. Organic and inorganic carbon levels were determined using Total Organic Carbon analyzer (TOC-V, Shimadzu, Japan). Total nitrogen (N) was estimated by Kjeldahl distillation method using Kjeltec Analyser (FOSS TECATOR, Sweden). Differences among soils were examined using SAS (SAS Institute Inc., United States).

### Direct Plating

This method was performed for isolating fungi from soil samples. Soil samples (0.1–0.15 g) were plated onto rose Bengal-amended 2.5% potato dextrose agar (Oxoid, England) plates using three replicates for each sample. Incubation was at 25°C for 3–7 days. Fungal colonies present on the incubation plates were subcultured for identification.

### Identification of Fungi

Fungal isolates were identified based on morphological characteristics under light microscope and sequences data. Fungal isolates were grown on PDA for 3–7 days. Then fungal isolates were preliminarily assigned to different genera based on the size and shape of spores and mycelia.

To confirm the identity of fungi, DNA was extracted from freeze dried mycelium using the protocol of Lee and Taylor (1990). The ITS region was amplified using the primer pair ITS1 and ITS4 (White et al., 1990) as explained by Al-Sadi et al. (2011). Additional loci (**β**-tubulin, Calmodulin, RNA polymerase II second largest subunit, Translation elongation factor 1-alpha) were used to identify the species of *Aspergillus, Cladosporium, Fusarium* and *Penicillium* using the primers and conditions detailed in literature (Carbone and Kohn, 1999; Samson et al., 2014). Purification and sequencing of PCR products were carried out at Macrogen, Korea. Sequences were aligned and improved using MEGA v.6 (Tamura et al., 2013). A maximum likelihood analysis was performed by using raxmlGUI v.1.3 (Silvestro and Michalak, 2012) for the isolates that belong to the kingdom fungi using the ITS region. The optimal ML tree search was conducted with 1000 separate runs, using the default algorithm. Bootstrap 50% majority-rule consensus trees were generated and the final tree was selected among suboptimal trees from each run by comparing likelihood scores under the GTRGAMMA substitution model. ITS sequences generated from the analysis were deposited in GenBank (**Table 2**).

**Table 2:** ITS GenBank accession numbers of fungal isolates detected in this study.

Fungal isolates	Accession number
*Aspergillus pachycristatus*	KY814690
*Aspergillus quadrilineatus*	KY814680
*Aspergillus quadrilineatus*	KY814684
*Aspergillus quadrilineatus*	KY814689
*Aspergillus rugulosus*	KY814676
*Aspergillus rugulosus*	KY814688
*Aspergillus rugulosus*	KY814687
*Cephaliophora* sp.	KY814682
*Chaetomium* sp.	KY814677
*Cladosporium tenuissimum*	KY814674
*Fusarium chlamydosporum*	KY814673
*Fusarium chlamydosporum*	KY814685
*Fusarium nygamai*	KY814686
*Fusarium solani*	KY814675
*Fusarium solani*	KY814679
*Fusarium solani*	KY814691
*Mortierella* sp.	KY814683
*Penicillium corylophilum*	KY814681
*Pythium aphanidermatum*	KY814678

### Pyrosequencing Analyses

DNA was extracted from 3 to 5 replicates from each soil sample according to the protocol of Volossiouk et al. (1995). A two-step process was used for the amplification of samples. Firstly, the forward (i5 and ITS1F) and reverse (i7 and ITS2aR) primers were constructed as described previously (White et al., 1990; Gardes and Bruns, 1993; Kazeeroni and Al-Sadi, 2016; Al-Balushi et al., 2017). The reaction mixtures and conditions for the first and the second PCRs were as per Al-Balushi et al. (2017). Checking of sequences was done using RDP ver 9 (Cole et al., 2009). Analysis and taxonomic classification was done using a distributed BLASTn.NET algorithm (Dowd et al., 2005) based on a 97% cut off. Fungi were classified based on trimmed taxa. The relative abundance for individual taxa was then determined after checking the percentage of sequences assigned to each fungal phylogenetic level.

## Results

### Soil Analysis

Soils differed in their properties (**Table 1**). The CM soil was sandy, while the soil from DE was loamy sandy. The pH was found to be alkaline in DE (7.8) and CM (8), while EC was significantly higher in DE (7.72) compared to CM (1.27) (*P* < 0.05; **Table 1**). The total inorganic carbon (TIC) and total organic carbon (TOC) concentrations were not significantly different between CM and DE (*P* > 0.05). The available N, P, and K concentration were significantly higher in the CM farming system compared to DE (*P* < 0.05; **Table 1**).

### Phylogenetic Analysis

The ITS alignment was used to represent the fungal species recovered from direct plating technique. The alignment comprised 68 strains (including the outgroup taxon *Allomyces reticulatus* and 18 isolates recovered in this study), and the manually adjusted dataset comprised 959 characters including gaps. A best scoring RAxML tree resulted with the value of Likelihood: -11745.862498 (**Supplementary Figure S1**). Based on the phylogenetic tree, 18 isolates from the present study belonged to *Ascomycota* phylum (classes *Dothideomycetes, Eurotiomycetes, Pezizomycetes* and *Sordariomycetes*), while the subdivision *Mucoromycotina* belonged to the phylum *Zygomycota*. Fungal classes were separated from each other with a very high bootstrap support (94–100%). Some of the isolates could not be matched with appropriate reference strains in GenBank, suggesting that some isolates could be new species or the sequence of their corresponding species are not available in GenBank.

### Evaluation of Fungal Diversity by Culture-Based Technique

**FIGURE 1:**
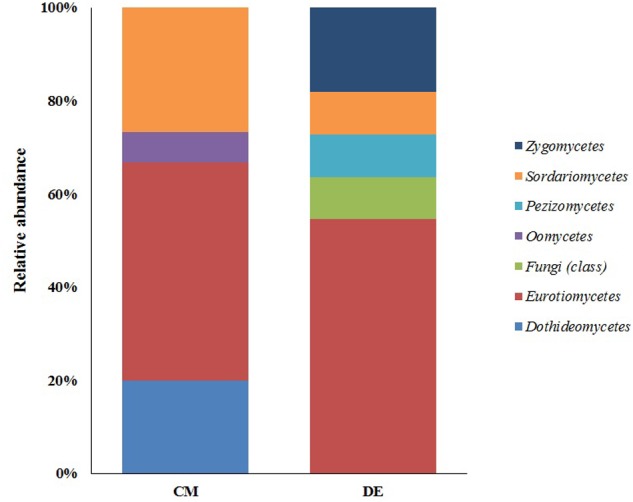
Class-level relative abundance of fungal communities in commercial farming (CM) and desert farming (DE) systems using culture-based technique.

**FIGURE 2:**
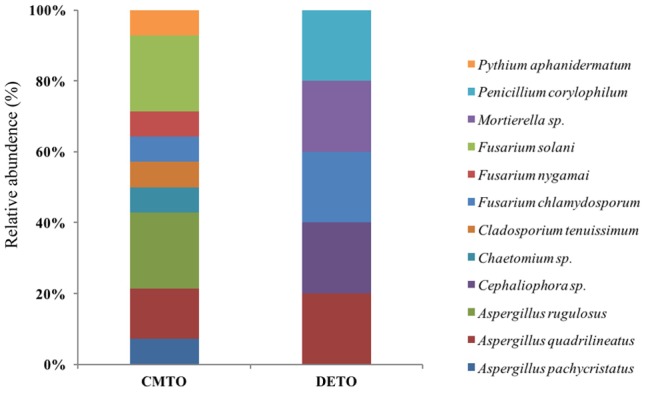
Species-level relative abundance of fungal communities in commercial farming (CM) and desert farming (DE) systems using culture-based technique.

*Ascomycota* was the most abundant phylum, present in both farming systems and *Oomycota* and *Zygomycota* were the other constituents. The phylum *Oomycota* was present only in CM while *Zygomycota* was present only in DM. In *Ascomycota*, soil samples from both farms presented a high relative abundance of *Eurotiomycetes* at class level (42.85% in CM, 40% in DE). This was followed by *Sordariomycetes* (42.85%) and *Dothideomycetes* (7.1%) in CM while *Sordariomycetes* and *Pezizomycetes* were found in the same level of abundance in the DE farming system (20%). *Dothideomycetes* and *Oomycetes* were unique classes in CM while *Zygomycetes* and *Pezizomycetes* were unique classes in DE (**Figure 1**). Totally eight genera were recovered from both farming systems and these were dominated by *Aspergillus* (**Figure 2**). A total of 12 fungal species were isolated from both farming systems. The most common species across two farming systems was *Aspergillus quadrilineatus*. *Cephaliophora* sp., *Mortierella* sp. and *Penicillium corylophilum* were only present in DE while *Chaetomium* sp., *Cladosporium tenuissimum, Aspergillus pachycristatus, A. rugulosus, F. nygamai, F. solani* and *Pythium aphanidermatum* were unique in CM. The Shannon values were 2.0 for soil from CM compared to 1.6 from DE (**Table 3**).

**Table 3:** Shannon-Wiener index of commercial farm (CM) and desert farm (DE) as determined by direct plating technique.

	Direct plating		Pyrosequencing	
	CM	DE	CM	DE
No. of phyla	2	2	5	3
No. of classes	4	4	8	9
No. of families	5	4	9	11
No. of genera	5	5	10	11
No. of species	9	5	15	11
Shannon Wiener index	2.0	1.6	1.4	1.9

### Evaluation of Fungal Diversity by Pyrosequencing Technique

**FIGURE 3:**
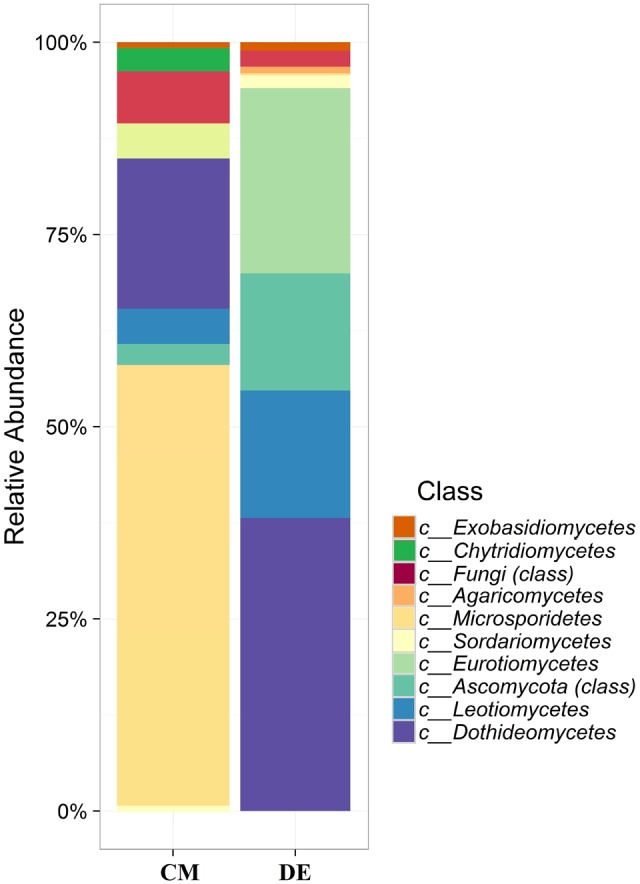
Class-level relative abundance of fungal communities in commercial farming (CM) and desert farming (DE) systems using pyrosequencing technique.

**FIGURE 4:**
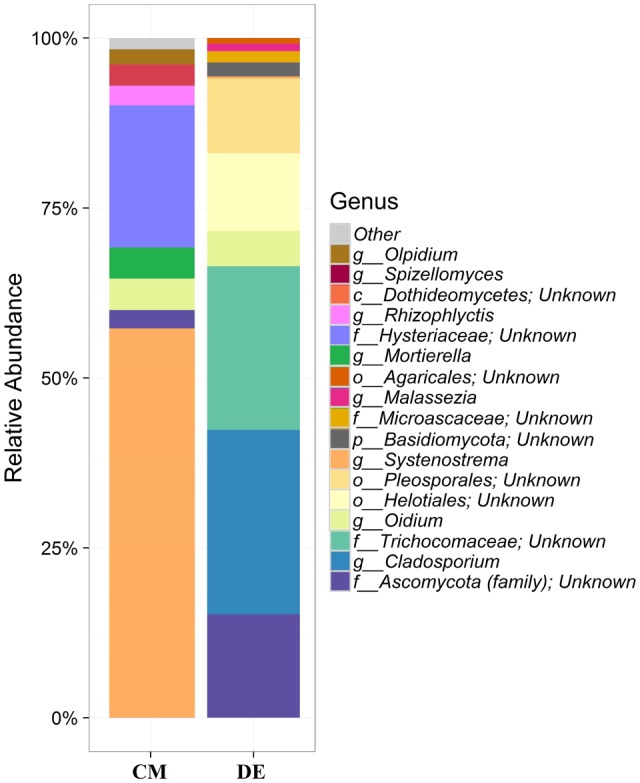
Generic-level relative abundance of fungal communities in commercial farming (CM) and desert farming (DE) systems using pyrosequencing technique.

Using a 97.0% similarity barcoding threshold, pyrosequencing showed that the majority of fungal taxa in CM was in the *Microsporidia* (60.26%), followed by *Ascomycota* (28.5%), *Chytridiomycota* (3.51%), *Basidiomycota* (0.77%) and *Zygomycota* (0.01%). All the *Microsporidia* belonged to a single class *Microsporidetes*. *Ascomycota* was distributed over classes *Leotiomycetes* (4.5%), *Dothideomycetes* (3%) and *Sordariomycetes* (1.5%). In DE over 95% of all OTUs belonged to the phylum *Ascomycota*, which was distributed in four classes; *Dothideomycetes* (38.07%), *Eurotiomycetes* (24.07%), *Leotiomycetes* (5.77%) and *Sordariomycetes* (4.04%). Another 23.73% could not be assigned to any classes and kept as Ascomycota *incertae sedis. Zygomycota* and *Chytridiomycota* were absent in the DE and *Microsporidia* contribution is in lesser amount (0.3%). The distribution of classes based on pyrosequencing is illustrated in **Figure 3**. The pyrosequencing approach yielded a total of 15 species in CM, with a Shannon value of 1.4 (**Figure 4** and **Table 3**). *Systenostrema alba* was the most dominant, comprising 60% of the total species in CM, followed by *Rhizina undulata* (17.56%) *Mortierella* sp. (4.17%) and *Oidium aloysiae* (4.04%). Pyrosequencing detected 11 fungal species in DE, with a Shannon value of 1.9 (**Table 3**). *Cladosporium* sp. (27%) and *Emericella nidulans* were the most abundant taxa, followed by *Trichocladium asperum* (12.8%), *Phoma gardeniae* (11%) and *Symbiotaphrina kochii* (10.94%).

### Direct Plating vs. Pyrosequencing

Direct plating and pyrosequencing methods were used in this study for estimating fungal abundance and diversity in two different farming systems of tomato. The water molds (*Oomycetes*) were not picked up by pyrosequencing and only detected by the culture-based method. On the other hand, *Basidiomycota, Chytridiomycota* and unicellular parasites *Microsporidias* were unable to be recovered using direct plating. Pyrosequencing detected more species compared to direct plating technique (**Table 3**).

## Discussion

Several studies using molecular techniques and cultivation-based methods have described the fungal communities present in different farming systems (Al-Sadi et al., 2015; Van Geel et al., 2015; Kazeeroni and Al-Sadi, 2016). These studies have shown that fungal communities present in each system vary with the soil physiochemical properties and the cropping systems (Huang et al., 2015; Thomson et al., 2015). Our results demonstrate that CM and DE soil are highly diverse in soil microbiota. In general, the fungal diversity in the CM farming system was high compared to the DE system. The presence of relatively high percentage of TOC and TIC in both farming systems may be favorable for the growth of most fungi. In addition, N, P and K levels are relatively high in CM soil and this is probably because of the addition of a certain amount of organic and chemical fertilizers to the soil. Thus, the application of fertilizers by growers could have contributed to creating differences in the available minerals in soils between the two farming systems and soil types (Grüter et al., 2017; Wang et al., 2017). Grantina et al. (2011) reported positive impact of the available potassium in soil on the total number of cultivable filamentous fungi (CFF) and on the fungal diversity. In another investigation, a negative impact of phosphorus was observed on species richness of fungi in soil (Huang et al., 2005). As suggested by Gyaneshwar et al. (2002), this could be due to variations in the number of phosphate solubilizing microorganisms in soil.

*Ascomycota* is the largest and widespread phylum of fungi and is abundant in soil and composts (Abed et al., 2013; De Gannes et al., 2013; Kazeeroni and Al-Sadi, 2016). They are considered important decomposers and causal agents of several soil-borne diseases. In the present study based on the culture-based technique, *Ascomycota* was dominant in both farming systems. *Eurotiomycetes* was identified as the dominant class in both CM and DE, mainly because it contains two of the most common fungal genera viz., *Penicillium* and *Aspergillus* in most of the ecosystems (Godinho et al., 2015; Yee et al., 2016). Many saprophytic fungi were detected in DE; whereas plant pathogenic fungi viz., *Cladosporium* sp., *Fusarium* spp. and *Pythium* sp. were detected in CM soil. In the present study, *Fusarium* that causes wilt disease in tomato was detected in CM soil samples. The prevalence of *Fusarium* in CM soil might be due to the potential ability of chlamydospores of *Fusarium* to survive in the soil for many years under harsh environmental conditions. Chellemi et al. (2012) demonstrated that repeated tomato cultivation increased the incidence of *Fusarium* wilt caused by *F. oxysporum* f. sp. *lycopersici* by 20% or more.

The mycoparasite, *Chaetomium* sp. was detected in the CE soil. However, other fungal biocontrol agents like *Trichoderma* spp. and *Gliocladium virens* were not detected in both the farming systems. The existence of these organisms in soil is crucial for suppression of damping off and Fusarial wilt diseases of tomato (Blaya et al., 2013; Guzmán-Valle et al., 2014). Hence, the soil health in both the farming systems has to be improved by incorporation of organic amendments and application of biopesticides. Some of the fungal isolates could not be identified to the species level, possibly because they are new species or they need more genes to be sequenced. Future studies may address the identity of these isolates.

Pyrosequencing revealed that 95% of the taxa in DE soil belonged to the *Ascomycota*, whereas 60% of the taxa present in CM soil belonged to the phylum *Microsporidia* (a group of spore-forming unicellular parasites) and the genus *Systenostrema.* This is in agreement with our previous findings that *Microsporidia* are one of the dominant phyla in soil of farming systems in Oman (Kazeeroni and Al-Sadi, 2016). *Microsporidia* are obligate, spore-forming, fungi-related, intracellular parasites that infect many vertebrates and invertebrates. Several species of microsporidia have been described as biocontrol agents and pathogens of several beneficial insects (Bjornson and Oi, 2014). For example, *Nosema pyrausta* is effective in controlling European corn borer (*Ostrinia nubilalis*) (Lewis et al., 2009). A formulation of *Paranosema locustae* is commercially available for biological control of rangeland grasshoppers (Bjornson and Oi, 2014). Several studies reported that *Microsporidia* are natural intracellular parasites of the nematodes including *Caenorhabditis* (Kaya et al., 1988; Troemel et al., 2008; Zhang et al., 2016). The *Microsporidia, Nematocida parisii* was reported as a natural intracellular pathogen of *Caenorhabditis elegans* (Troemel et al., 2008). Zhang et al. (2016) described six new species in the *Nematocida* genus that are capable of infecting *Caenorhabditis elegans*.

In general, fungal species diversity was higher with the pyrosequencing than the culture-based method. These dissimilarities are to be expected and are not surprising since many of the fungi are not cultivable. Some studies showed that approximately 1% of the total microbes could be detected by culture-based methods (Sugiyama et al., 2010). On the contrary, some fungi can easily be cultured even they are present in small quantities. In addition, the high temperature in the Omani desert, which sometimes exceeds 50°C in summer, could have affected fungal diversity in soil by killing or suppressing several fungal species that are heat sensitive (Abed et al., 2013; Classen et al., 2015; Costa et al., 2015). This in turn could have affected the number of fungal species recovered from soil by culture-dependent methods. Also the absence of *Chytridiomycota* and *Basidiomycota* in culture-based method could have been because they either need specific media or their presence was limited as evidenced by pyrosequencing analysis (only 3.51% and less than 0.77% of the total soil population, respectively) (Gleason et al., 2007; Yee et al., 2016). Therefore, the detection of the precise diversity of fungi in a habitat using culture-based techniques is still challengeable. Pyrosequencing recovered more species that were not revealed by the culture-based method, implying that this approach will speed up the detection of very rare fungal species (Huang et al., 2015; Liu et al., 2017). However, pyrosequencing still has some limitations in describing fungal diversity. Future studies should investigate if using other media in addition to PDA and also other genes for pyrosequencing could help recover more fungal species and reduce the existing gap between pyrosequencing and culture based techniques.

## Conclusion

This study provided evidence that farming systems strongly influence the composition of soil fungal communities. It is surprising to note that a few soil fungi that were detected by direct culturing method could not be detected by pyrosequencing. More research is required by using different soil DNA extraction procedures. Culturing fungi by using multiple nutrient media might result in the isolation of additional fungi from soil. Viability of fungal communities in soil needs to be considered when assessing their diversity in a farming system. One of the major drawbacks in PCR-based methods is their inability to discriminate between nucleic acids from viable and dead cells. The DNA extracted from dead cells can also serve as a template in PCR amplification. To overcome such issues, viability PCR using propidium monoazide (PMA) that differentiate nucleic acids from live and dead cells (Cangelosi and Meschke, 2014) has to be tested.

## Author Contributions

AA-S, EK, SM, and HA-M planned the experiment. EK and HA-M conducted the experiment, AA-S, EK, SM, and VR analyzed data, EK, AA-S, SM, VR, and HA-M wrote the manuscript. All authors approved the manuscript.

## Conflict of Interest Statement

The authors declare that the research was conducted in the absence of any commercial or financial relationships that could be construed as a potential conflict of interest.

## Supplementary Material

FIGURE S1 Phylogenetic analysis of fungi recovered from direct plating based on ITS data set. The tree is rooted with *Allomyces reticulatus* (*Blastocladiomycota*). RAxML bootstrap values higher than 50% are given above or below the nodes. The isolates from present study are in bold.
