# Supplementary figures and images for: Fungal Diversity in Tomato Rhizosphere Soil under Conventional and Desert Farming Systems
Source: Front Microbiol. 2017 Aug 2;8:1462. doi: 10.3389/fmicb.2017.01462 (PMC5539375; doi:10.3389/fmicb.2017.01462)

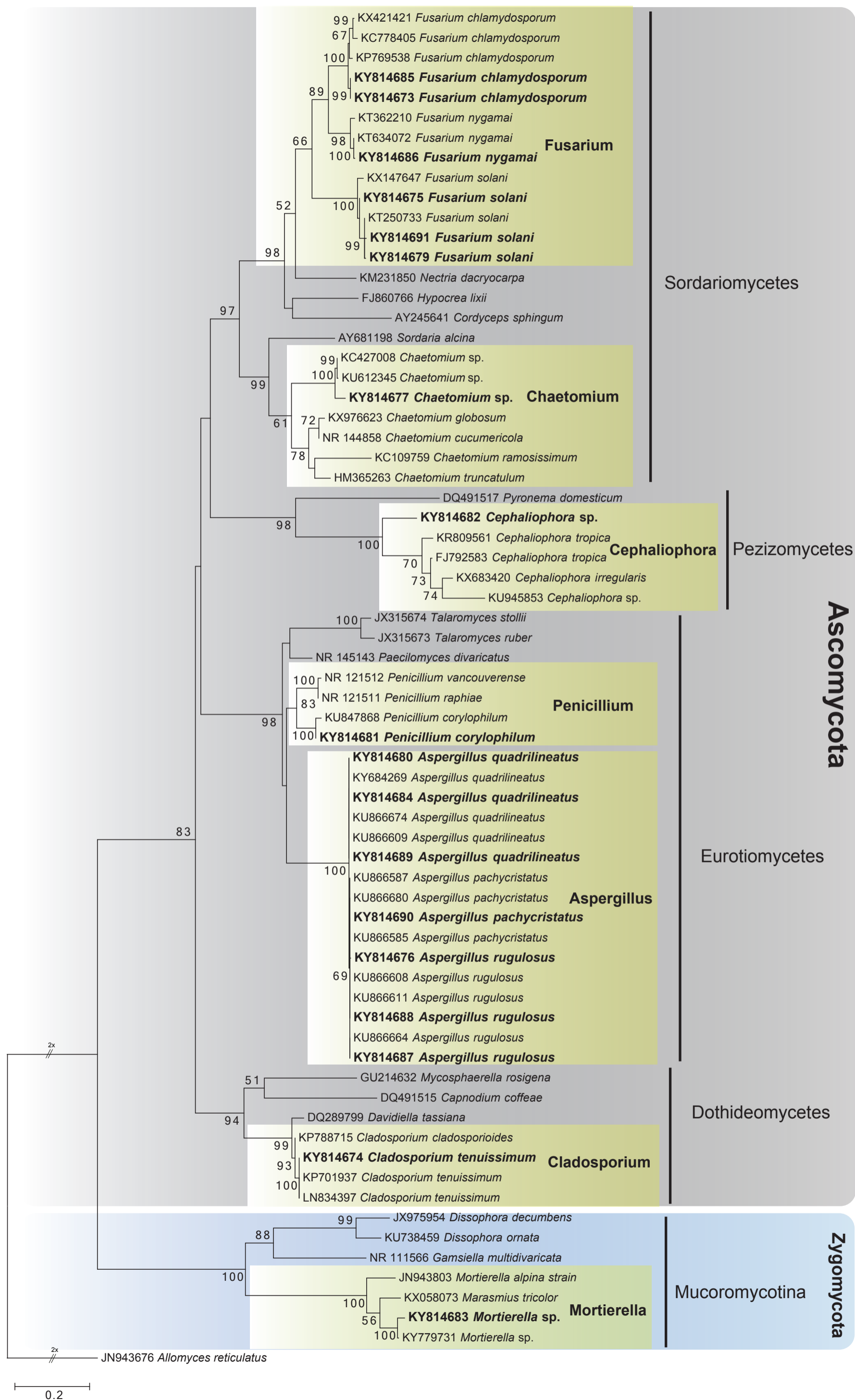

Supplement: FIGURE S1 — Phylogenetic analysis of fungi recovered from direct plating based on ITS data set. The tree is rooted with Allomyces reticulatus (Blastocladiomycota). RAxML bootstrap values higher than 50% are given above or below the nodes. The isolates from present study are in bold. [file Image_1.PDF]
